# Assessing the benefit of acoustic beamforming for listeners with aphasia using modified psychoacoustic methodsa)

**DOI:** 10.1121/10.0002454

**Published:** 2020-11-24

**Authors:** Sarah Villard, Gerald Kidd

**Affiliations:** Department of Speech, Language, and Hearing Sciences, Boston University, 635 Commonwealth Avenue, Boston, Massachusetts 02215, USA

## Abstract

Acoustic beamforming has been shown to improve identification of target speech in noisy listening environments for individuals with sensorineural hearing loss. This study examined whether beamforming would provide a similar benefit for individuals with aphasia (acquired neurological language impairment). The benefit of beamforming was examined for persons with aphasia (PWA) and age- and hearing-matched controls in both a speech masking condition and a speech-shaped, speech-modulated noise masking condition. Performance was measured when natural spatial cues were provided, as well as when the target speech level was enhanced via a single-channel beamformer. Because typical psychoacoustic methods may present substantial experimental confounds for PWA, clinically guided modifications of experimental procedures were determined individually for each PWA participant. Results indicated that the beamformer provided a significant overall benefit to listeners. On an individual level, both PWA and controls who exhibited poorer performance on the speech masking condition with spatial cues benefited from the beamformer, while those who achieved better performance with spatial cues did not. All participants benefited from the beamformer in the noise masking condition. The findings suggest that a spatially tuned hearing aid may be beneficial for older listeners with relatively mild hearing loss who have difficulty taking advantage of spatial cues.

## I. INTRODUCTION

It is common for individuals with aphasia—i.e., language impairment resulting from stroke or other neurological injury/disease—to report difficulty understanding speech in noisy environments. The challenge of listening to target speech while ignoring or filtering out background noise, known as the “cocktail party problem” [Cherry (1953); see Middlebrooks *et al.* (2017) for a series of recent reviews], has high relevance for everyday communication, as real-world conversations often take place in settings that are acoustically complex. While the majority of past research on receptive speech processing in persons with aphasia (PWA) has focused on auditory language comprehension in quiet settings, several recent studies have directly investigated the ability of persons with aphasia (PWA) to selectively attend to and understand speech in the presence of auditory maskers [e.g., Rankin *et al.* (2014) and Villard and Kidd (2019)]. These studies have provided evidence that PWA—even, in some cases, PWA with milder aphasia types thought to be characterized primarily by expressive language deficits—require higher target-to-masker ratios (TMRs) than do age-matched controls in order to successfully understand target speech. These laboratory-based findings validate the anecdotal reports from PWA that background sounds can present major challenges in their ability to understand conversational partners in everyday situations.

While it is not yet clear precisely why PWA encounter difficulty in multi-talker environments, there is reason to believe that this difficulty could be due to impairments in cognitive abilities such as selective attention. Aphasia has historically been defined as a disorder that solely affects language abilities, leaving cognitive abilities intact. However, the past several decades have seen an increasing focus in the aphasia literature on impairments in cognitive areas including attention (Hula and McNeil, 2008; Murray, 2000, 2012; Villard and Kiran, 2017), which may be relevant in cocktail party situations where listeners must selectively attend to a target speech stream while ignoring maskers. The observation that PWA may encounter difficulty understanding speech when background sounds are present also gives rise to the question of what types of strategies or approaches might be effective in helping PWA to improve communication in complex sound environments. The identification or development of such strategies, whether rehabilitative or compensatory in nature, is a goal of both clinical significance and practical importance. The primary goal of the current study is to investigate whether a compensatory technique involving acoustic beamforming could improve PWA performance on a masked speech recognition task.

A secondary but related goal of the current study is to explore a possible approach to modifying psychoacoustic methods for use with PWA. Typically, determination of whether a particular rehabilitative approach or compensatory technique is effective for a group of listeners would involve the administration of certain standard speech recognition or psychoacoustic measures. However, it is generally acknowledged that a variety of factors associated with aphasia may make it challenging to obtain valid and reliable measurements from PWA using these tasks, which were developed to test listeners without known cognitive-linguistic impairments. These factors include not only the language deficits that characterize aphasia, but also associated deficits in areas such as reading and verbal working memory. In order to ensure accurate assessment of the benefit of potential strategies for improving speech intelligibility in PWA participants, careful modifications of typical psychoacoustic methods may need to be devised and implemented. Therefore, while the current study's primary focus is on the question of whether a compensatory auditory prosthesis utilizing acoustic beamforming could provide a benefit to some PWA in cocktail party listening situations, it also examines an approach for adapting typical psychoacoustic methods for the purpose of obtaining accurate measurements of speech recognition abilities in PWA.

### A. Energetic and informational masking in aphasia

In measuring the effects of masking and considering possible strategies for improving masked speech recognition, it is important to distinguish between the effects of two broadly defined types of masking: *energetic* and *informational*. Energetic masking (EM) refers to reduced neural representation of target sounds due to time-frequency overlap with masker sounds. That is, if the masker energy exceeds the target energy in a given time-frequency region, then the masker likely will dominate the peripheral neural representation of the combined stimulus in that region, making the target difficult or impossible to detect or to identify [e.g., Conroy *et al.* (2020)]. In that case, performance is adversely affected because the neural representation of the target is “data limited”; i.e., sufficient information from the target source does not propagate up the auditory pathways. However, adequate peripheral neural representation of the target does not ensure the absence of masking. Certain types of listening situations have been found to produce substantial levels of informational masking (IM), or additional masking beyond any EM present in the signal [e.g., Brungart *et al.* (2001), Freyman *et al.* (2004), Calandruccio *et al.* (2013), Holmes *et al.* (2018); reviews in Kidd *et al.* (2008) and Mattys *et al.* (2012)]. IM is thought to result from limitations in the listener's later-stage central processing capabilities and tends to be high in speech-on-speech masking situations where the masker and target are difficult to separate and are easily confused despite adequate audibility of the target [see Kidd and Colburn (2017) for a review]. Susceptibility to IM has been shown to vary considerably from listener to listener, even among young adult listeners with normal hearing, possibly due to inter-individual variability in higher-level cognitive skills such as attention or working memory [e.g., Clayton *et al.* (2016) and Oberfeld and Kloeckner-Nowotny (2016)].

When considering how masking affects speech recognition in PWA, IM may be of particular interest because aphasia produces cognitive-linguistic impairments but does not have any known effect on peripheral auditory function. Our recent study on the respective effects of EM and IM in PWA found that when simple target sentences were masked by speech-shaped, speech envelope-modulated noise—a high-EM, low-IM condition—PWA and age-matched controls performed similarly. However, when the same sentences were masked by intelligible speech—a high-IM, low-EM condition—PWA required significantly higher TMRs in order to comprehend the target sentences (Villard and Kidd, 2019). These results suggest that PWA experience a particular difficulty in perceptually segregating target speech from masker speech and/or selectively attending to target speech. Because the hearing profiles were similar between the two groups in that study, and because only very simple experimental stimuli were presented, the observed group difference likely was not attributable to peripheral factors or to underlying language comprehension deficits. As susceptibility to IM in speech-on-speech masking conditions is thought to be related to higher-level cognitive processes [e.g., Swaminathan *et al.* (2015) and Clayton *et al.* (2016)], it is hardly surprising that high-IM conditions pose problems for PWA, who by definition have damage to central processing areas.

The finding that PWA may be highly susceptible to the effects of IM could have a variety of implications for these listeners. To begin with, it suggests that PWA may struggle to understand speech in everyday situations where multiple conversational streams are audible; examples might include the family dinner table, a holiday party, the intermission during a play or concert, or even the checkout line at a supermarket. Difficulty understanding a conversational partner in settings like these could adversely affect social relationships, community participation, and quality of life in PWA. Additionally, it is plausible that difficulty filtering out background talkers or other distracting sound sources could mitigate the benefits of language therapy in PWA. Particularly in the earlier stages of recovery, PWA often undergo language therapy in medical settings, many of which may contain substantial background sounds, including the voices of medical personnel, other patients, and visitors, as well as voices from sources such as intercoms and televisions. These auditory environments could therefore contain a substantial amount of IM (Pope, 2010; Pope *et al.*, 2013). Because background sounds in real-world settings often are difficult to control or to modify, the development of techniques to accurately measure the effects of auditory masking in such environments on PWA, as well as strategies to help PWA better understand target speech, could make a substantial difference in the everyday lives of individuals living with aphasia.

Furthermore, although damage to central processes may be a driving factor behind the challenges faced by PWA in complex listening situations, the possible impact of peripheral factors on the ability to understand speech and the effects of masking is also essential to consider in this population. Aphasia is most common in older individuals, and there has been an increasing recognition of the possibility that many PWA may demonstrate some degree of age-related sensorineural hearing loss (SNHL) in addition to their language deficits (Formby *et al.*, 1987; Silkes and Winterstein, 2017; Zhang *et al.*, 2018). The challenge of understanding and addressing the respective contributions of peripheral and central factors to masking susceptibility in individual PWA therefore complicates both the characterization of this problem and the development of possible strategies to address it.

### B. Acoustic beamforming as a possible compensatory strategy for listeners with aphasia

Because the literature on auditory masking in PWA is still quite limited, especially with respect to elucidation of the relative influences of EM and IM, little is currently known about how speech recognition abilities in complex acoustic environments can be improved in this population. A fundamental question in beginning to investigate possible strategies for improving communication is whether a compensatory/prosthetic approach (such as a hearing aid or other amplification system) or a rehabilitative approach (such as auditory training) would be most effective. While commercially available hearing aids designed for listeners with SNHL could provide assistance to PWA in some situations (e.g., improving audibility if hearing loss is present), the potential benefit of standard hearing aids is limited in situations comprising multiple competing talkers in part because they amplify the maskers as well as the target, often providing only modest improvements in TMR. Hearing aids or other amplification systems that include a strong directional component, however, can be more useful in complex auditory environments because they may provide amplification that emphasizes a target source location.

Acoustic beamforming is a highly directional amplification approach that has been found to be effective in laboratory-based studies of speech-on-speech masking in listeners with SNHL [e.g., Kidd *et al.* (2015)]. The beamforming technology used in past work from our group [e.g., Kidd *et al.* (2013), Kidd *et al.* (2015), Best *et al.* (2017), and Roverud *et al.* (2018)] consists of an array of spatially distributed, omni-directional microphones worn on the head of a human listener—or, more commonly, the beamforming algorithm is implemented for headphone-based presentation using impulse responses measured while the array is positioned on the KEMAR manikin [see Kidd (2017) for details]. When implemented, the beamformer effectively attenuates sounds originating from locations that are off-axis from a designated target location (usually either directly in front of the listener or at an azimuth specified by eye gaze) and subsequently presents the combined target/masker signal to the listener via a single channel (i.e., monotic or diotic presentation). While the single channel output signal lacks the binaural spatial cues available to listeners in naturalistic, unaided listening situations, its key advantage is an improved TMR that can help boost listener performance on a speech recognition task. Thus, the perceptual segregation of target and masker by relative level is enhanced by the single-channel beamformer; however, this enhancement comes at the cost of the loss of perceptual segregation cues resulting from interaural differences in target and masker waveforms.

In general, the beamforming approach used in the present study has been shown to provide significant benefits for masked speech recognition when there is sufficient spatial separation between target and maskers under certain conditions for both normal hearing (NH) and SNHL listeners (Kidd, 2017; Kidd *et al.*, 2015), and more recently in cochlear implant users (Yun *et al.*, 2019). In particular, implementation of the beamformer was found to result in significantly lower (better) speech reception thresholds (SRTs) for both NH and SNHL listeners in a high-EM listening condition where maskers consisted of speech-shaped, speech envelope-modulated noise that was spatially separated from the target (Kidd, 2017). However, in high-IM listening conditions where maskers comprise intelligible speech and are spatially separated from the target, the effect of the beamformer (i.e., whether a benefit is observed and the magnitude of the benefit) is more complex and depends on factors such as the degree of hearing loss and, potentially, age [e.g., Gallun *et al.* (2013)]. In the Kidd *et al.* (2015) study, only the listeners with the poorest performance under “natural” spatial hearing conditions (i.e., simulated by KEMAR head-related transfer functions) obtained a significant benefit whereas all of the cochlear implant subjects in Yun *et al.* (2019) obtained a significant benefit from the beamformer. In some cases, NH listeners achieved significantly better SRTs *without* the beamformer using natural binaural cues in unaided listening (Kidd *et al.*, 2015). These results also suggested that person-to-person performance varied substantially within both groups—a finding that is typical for high-IM conditions [e.g., Kidd and Colburn (2017)].

Prior to the current project, little was known about the extent to which beamforming might provide a benefit to PWA listeners. Importantly, PWA differ from populations in which the beamformer has previously been tested, in that they exhibit known cognitive-linguistic deficits that are central in origin and, as a result, the challenges they encounter in cocktail party listening situations could be rooted in somewhat different factors. Additionally, PWA tend to be older than many of the listeners in which beamforming has previously been tested, introducing the possibility of age-related cognitive differences, as well as possible differences related to age-related hearing loss. Given these differences, directly measuring the effect of beamforming in PWA listeners will provide valuable information about whether it could be an appropriate compensatory aid for use in this population.

Additionally, assessing the benefit of acoustic beamforming in PWA may provide information about the factors that facilitate or hinder the ability of PWA to understand speech in complex acoustic environments. While our previous work has found that PWA perform more poorly than controls under a condition where maskers consist of intelligible speech spatially separated from the target (Villard and Kidd, 2019), the precise reason(s) for this are not yet known. Because beamforming provides the listener with both a distinct advantage (an improved TMR) and a distinct disadvantage (removal of binaural spatial cues), relative to a natural listening condition, it may allow us to learn more about what drives PWA performance. For example, if the reason PWA perform more poorly under naturalistic listening conditions involving binaural spatial cues is simply that they have difficulty taking advantage of those cues to separate speech streams, then beamforming—which removes these cues and replaces them with an improved TMR delivered through a single channel—might be expected to provide a notable benefit for PWA. However, if the poorer performance observed in PWA arises not from difficulty utilizing binaural spatial cues but rather from difficulty with higher level cognitive skills involved in disentangling intelligible speech streams, then beamforming might offer somewhat less of an advantage in masked speech recognition (although, it should be noted, an improved TMR could also result in somewhat of a reduced cognitive processing load for the listener).

### C. Modifications of psychoacoustic methods for listeners with aphasia

The process of assessing the benefit of beamforming in PWA listeners is complicated by the fact that the methodology used in our previous work on beamforming—and indeed, in many typical psychoacoustic experiments or standard clinical procedures (e.g., verbal word recognition/repetition tasks) assessing speech intelligibility—may not be appropriate for testing PWA (Zhang *et al.*, 2018). Because addressing questions related to speech intelligibility typically necessitates extensive measurements (i.e., many repetitions across numerous conditions), the psychoacoustic procedure of choice often involves the use of closed-set, forced-choice, matrix-style speech identification methods designed to minimize the effects of learning on performance. Such methods afford the advantage that the limited set of items can be vetted in advance to assure familiarity to the participant and, because the items are selected at random on each trial from a closed set, the concerns about prior exposure to the specific test items (e.g., a limited number of complete sentences available, limited numbers of talkers available, etc.) that may confound multiple repetitions of lists of open set materials are moot [e.g., Webster (1983)]. In such procedures, performance typically is evaluated by presenting auditory stimuli throughout a sequence of trials, with a response made after each stimulus/trial often via a graphical user interface (GUI) by mouse-clicking or touching the response alternative on the computer screen. The GUI usually contains a list, or lists, of words from which the listener is asked to select the target words one by one. For example, a typical target sentence might contain five (or more) words, and the listener might be presented with a large GUI containing five (or more) lists of response options, one for each word in the sentence (or, alternatively, a series of GUIs that appear on the screen one at a time, one for each word in the target sentence).

While these matrix-style experiments offer a number of important advantages, they may cause unintended experimental confounds if administered to listeners with known cognitive-linguistic impairments. To begin with, while these tests are intended to measure the listener's speech recognition abilities, the response selection process presents a number of additional demands, as listeners must efficiently read and/or scan through multiple lists of response options while continuing to hold the target sentence in memory. Such demands are not thought to be particularly taxing for listeners in the general population. However, because many PWA exhibit deficits in reading, scanning, and working memory, these response-related demands could present a substantial additional challenge, resulting in artificially reduced performance for PWA despite adequate recognition of target speech. Additionally, many PWA have impaired verbal repetition skills, which may make it difficult or impossible for them to use common strategies such as verbal rehearsal to assist them with their responses. Therefore, the development of approaches to control/minimize these confounds so that psychoacoustic measures may be used to obtain valid measurements of speech intelligibility in PWA listeners is a key element of assessment.

### D. Aims of the current study

The current study had three aims. The first and primary aim was simply to determine whether acoustic beamforming could provide a benefit for PWA in understanding speech in acoustically complex environments. Although prior work had demonstrated that beamforming can provide a benefit for listeners with SNHL, it could not be assumed that PWA would receive a similar benefit, despite the evidence suggesting that persons with SNHL and PWA both experience difficulty understanding speech in complex acoustic environments [e.g., Kidd *et al.* (2019) and Villard and Kidd (2019)]. This is because the factors underlying these two groups' difficulties almost certainly differ, particularly in terms of whether the limitation on performance is predominantly peripheral or central in origin. For listeners with SNHL, the poor performance for spatially separated speech and masker sources likely is due to a degraded peripheral representation of the sounds which is known to increase EM [e.g., Arbogast *et al.* (2005), Marrone *et al.* (2008), and Best *et al.* (2012)], though for older listeners with SNHL, cognitive factors could also be at play (Gallun *et al.*, 2013). The single channel beamformer eliminates the perception of spatial separation of sources that occurs through normal binaural hearing and therefore eliminates the benefits of using interaural differences to enhance source segregation. Balanced against that loss of spatial perception is the increase in signal-to-noise ratio (S/N) from the spatial tuning of the beamformer. In order to solve the source segregation problem using the beamformer in a multiple talker sound field, the listener must rely on the improvement in relative level of the target source, as well as the different voice characteristics, to disentangle the talkers. Because PWA presumably do not have the same peripheral deficit as SNHL (e.g., reduced frequency and time resolution due to sensorineural pathology), the improvement in S/N from the beamformer may not compensate for the loss of the percept of spatial segregation of sounds to a similar degree and thus may not provide the same benefit for PWA as for SNHL, or may do so under some masking conditions and not others (e.g., high EM vs high IM). Notably, because aphasia is more common in older individuals, many PWA may also have some age-related peripheral hearing loss, and thus may also experience increased EM. However, the limited evidence available has shown that additional, centrally based processing problems likely are present (Villard and Kidd, 2019), potentially resulting in a mixed peripheral-central processing deficit. As discussed earlier, the extent to which PWA can utilize various sound source segregation cues currently is not known and the extent to which enhancing specific cues—such as segregation of competing speech sounds by level from a single-channel beamformer—is beneficial also is not clear. This study therefore examined the extent to which acoustic beamforming could improve speech recognition in PWA.

The second aim of the study was to examine the effect of acoustic beamforming as a front-end signal processing strategy on masked speech recognition in a group of controls who were age- and hearing-matched to the PWA listeners, and to compare this effect to that seen in the PWA group. Because PWA and age- and hearing-matched (i.e., audiometrically similar) controls would be assumed to have similar peripheral hearing abilities, but to differ in central processing abilities, a comparison of the effect of beamforming on these two groups could help to clarify aspects of the central processing difficulties observed in PWA. Thus, the potential benefit of the beamformer was examined under both high-EM, low-IM conditions and high-IM, low-EM conditions, in both PWA and controls. Our expectation was that differences between the PWA and control listeners, if present, would be more apparent in a high-IM speech-on-speech masking condition than a high-EM speech-on-noise masking condition because aphasia is a central nervous system disorder.

The third and final aim of the study was to assess the feasibility of modifications of standard psychoacoustic/speech recognition methods for use with PWA. As discussed above, typical psychoacoustic methods using matrix-style sentences that require participants to read through word lists in order to respond may present challenges/confounds for PWA participants. In our previous study, we sought to bypass the majority of these confounds by using a very small response set containing only highly imageable nouns, with pictures as response options instead of written words (Villard and Kidd, 2019). Although this approach was effective for the purposes of that study, it did have some limitations, particularly with respect to the types of words that could easily be represented by graphical images. The current study, therefore, took a different approach to adapting the demands of the task to the abilities of the participant. Here we retained the speech matrix test used previously in studies of the benefits of beamforming for NH and SNHL participants (Kidd *et al.*, 2015), a test that depends on the use of written words as response options. However, modifications of sentence length and the number of available response items in each syntactic category were made to accommodate the abilities of individual PWA. Because the PWA population is quite heterogeneous, with different individuals displaying different degrees of difficulty with tasks such as reading and working memory, we chose to determine the extent of these modifications individually for each participant, using a combination of rule-based decision-making and clinical judgment,^1^ as outlined in Sec. II. The goal of this approach was to employ stimuli that were closer to those used in previous psychoacoustic experiments examining the effects of acoustic beamforming, while taking into consideration each PWA listener's specific limitations. Importantly, this effort has implications not only for assessing the benefit of acoustic beamforming in PWA but potentially also for the investigation of speech recognition in complex listening conditions in other populations with impaired language and/or cognition who cannot reliably be tested using standard methods.

## II. METHODS

### A. Participants

A total of ten listeners served as participants in this experiment. An eleventh participant was dismissed after failing to meet the minimum performance criteria for participation (as explained further below). Of the ten remaining participants, five demonstrated aphasia resulting from a stroke in the language-dominant hemisphere (as did the eleventh participant). All participants with aphasia were in the chronic stage of recovery, meaning that their stroke had occurred more than 12 months prior to participation. Participants were recruited through existing participant databases at Boston University and through online advertisements. All participants demonstrated visual acuity that was adequate for task completion.

Each PWA participant's aphasia type and aphasia severity were identified using Part 1 of the Western Aphasia Battery-Revised (WAB-R) (Kertesz, 2007), a standardized language measure. WAB-R results indicated that two participants (PWA1 and PWA6) exhibited Broca's aphasia, a non-fluent aphasia type characterized by notable difficulty with word-finding and sentence formulation. The remaining four (PWA2, PWA3, PWA4, and PWA5) exhibited anomic aphasia, a fluent aphasia type characterized primarily by milder expressive word-finding difficulty. The WAB-R also provides Aphasia Quotients (AQs) indicative of overall aphasia severity. These scores suggest that PWA1 and PWA6 each exhibited a moderate aphasia, while the remaining four PWA participants exhibited a mild aphasia. In order to collect information about participants' selective attention abilities, the Map Search and Elevator Counting with Distraction subtests of the Test of Everyday Attention (TEA) (Robertson *et al.*, 1994) were also administered. The Map Search task requires participants to quickly locate as many instances as possible of a specific visual symbol on a visually cluttered map, and the Elevator Counting with Distraction task requires participants to attend to, count, and report the number of target tones heard while ignoring non-target tones (participants were offered the use of a number line during each response period to point to their answer rather than verbalizing it, if they preferred). Please see Table I for information on standardized test results in PWA participants, as well as possible score ranges.

**TABLE I.** Standardized testing results for PWA participants. AQ range: 0–100; lower score indicates a greater deficit. An AQ of 51–75 is considered to indicate moderate aphasia; an AQ of 76 and above is considered to indicate mild aphasia. Map search range: 0–80; lower score indicates poorer performance. Elevator Counting w/Distraction task range: 0–10; lower score indicates poorer performance.

	Aphasia type	WAB AQ	TEA: Map search (2 min)	TEA: Elevator counting w/ distraction
PWA1	Broca's	63	74	9
PWA2	Anomic	96	44	9
PWA3	Anomic	96	56	1
PWA4	Anomic	98	35	2
PWA5	Anomic	90	23	6
PWA6	Broca's	59	21	2

The remaining five participants reported no history of stroke, brain injury, or other neurological event/disease and served as age- and hearing-matched controls. Each control participant was matched with one PWA participant, resulting in five pairs of participants. The age difference between the PWA participant and control participant within a given matched pair was no more than three years. Efforts were also made to match pairs according to hearing profile; however, matching by age was prioritized. Because of the challenge of matching across two parameters, one pair (PWA5 and C5) had somewhat mismatched hearing profiles. Please see Table II for details on the five matched pairs.

**TABLE II.** Information on matched PWA-control pairs. 4 F-PTA = four-frequency pure tone average, or the average of pure tone thresholds at 500 Hz, 1 kHz, 2 kHz, and 4 kHz.

	Sex	Age	4F-PTA (left ear)	4F-PTA (right ear)
PWA1	M	54	15.0	12.5
PWA2	M	53	25.0	17.5
PWA3	F	61	9.4	8.8
PWA4	F	56	15.6	13.8
PWA5	M	67	32.5	32.5
	*Average:*	*58.2*	*19.5*	*17.0*
C1	F	56	11.0	16.3
C2	F	56	21.0	28.8
C3	M	62	9.4	8.8
C4	M	56	6.9	5.0
C5	M	64	11.3	8.8
	*Average:*	*58.8*	*11.9*	*13.5*

**FIG. 1.**
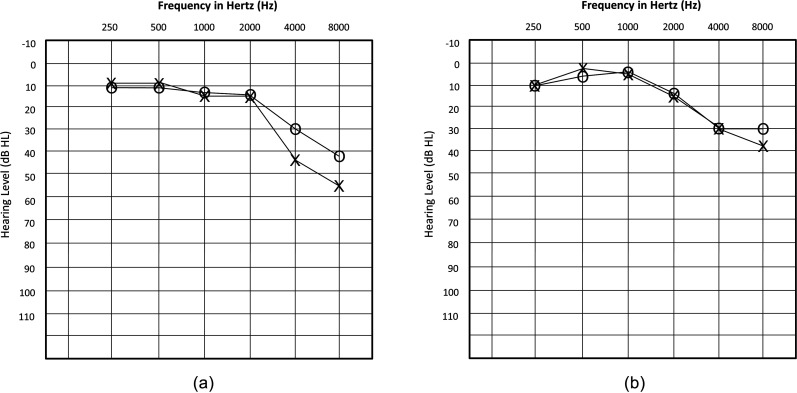
(a) Average pure tone audiograms for PWA. (b) Average pure tone audiograms for controls.

All participants completed pure tone hearing testing in each ear, and all participants demonstrated some degree of hearing loss [see Figs. 1(a) and 1(b) for average audiograms for PWA and controls]. This loss of sensitivity was generally greater at higher frequencies and was believed (based on participant report) to have been acquired in adulthood. No participant with aphasia reported any perceived link between their stroke history and hearing sensitivity. Hearing loss was relatively mild across participants, and no participants reported current or past use of hearing aids. This study was overseen by the Institutional Review Board at Boston University.

### B. Experimental stimuli

Auditory stimuli consisted of recordings of 40 single words drawn from an 8 × 5 matrix (8 names, 8 verbs, 8 numbers, 8 adjectives, and 8 objects; see Table III) that has been used in a number of previous psychoacoustic experiments involving speech masking (Kidd *et al.*, 2008; Swaminathan *et al.*, 2015; Clayton *et al.*, 2016), including experiments on beamforming (Kidd *et al.*, 2015). Eight different recordings of each word were used in the study, each one spoken by a different female talker (i.e., each of eight talkers recorded the entire set of words), for a total of 320 total single-word recordings. Visual stimuli consisted of columns of typed words presented on a GUI on a computer screen. Stimuli were presented, and data were collected, using custom software in matlab (MathWorks, Inc., Natick, MA).

**TABLE III.** Full experimental matrix.

Names	Verbs	Numbers	Adjectives	Objects
Bob	bought	two	big	bags
Jane	found	three	cheap	cards
Jill	gave	four	green	gloves
Lynn	held	five	hot	hats
Mike	lost	six	new	pens
Pat	saw	eight	old	shoes
Sam	sold	nine	red	socks
Sue	took	ten	small	toys

### C. Individualized modifications and frequency-specific gain

In previous experiments in our laboratory, participants typically have been presented with auditory signals consisting of sentences having the structure <name> <verb> <number> <adjective> <object> (e.g., “Jane sold six new bags”), followed by five lists of words printed on the computer monitor, each consisting of the full set of words in a given syntactic category (e.g., Kidd *et al.*, 2008). Participants have been instructed to select each of the target words—usually by mouse clicking the selection from a GUI—from the available options, one by one. As discussed earlier, however, this approach presents substantial working memory, reading, and scanning demands to the participant, in addition to the primary task (speech recognition). The ability of individual PWA to quickly and accurately record their response may vary substantially, possibly interacting with their ability to perform the primary task. In order to reduce the potential for aphasia-related deficits unduly influencing the results (i.e., causing confounds) while preserving the overall structure and essential nature of the task, therefore, two experimental parameters—sentence length and number of response options presented per column—were modified individually for each PWA participant.

Prior to beginning the experiment, the first author, a certified speech-language pathologist with substantial experience evaluating and treating PWA in both clinical and research settings, worked with each PWA participant to determine an optimal sentence length and number of response options per column. The two guiding principles for determining the optimal modifications for a given PWA participant were that (a) both parameters should be maximized to the extent possible, but that (b) the participant should still be able to achieve ceiling performance on the modified task in a “quiet” condition (no maskers present). To determine experimental parameters that fulfilled both of the guiding principles, a series of masker-free practice trials was presented to the participant, and a combination of clinical judgment and trial and error was used to adjust the experimental parameters until a challenging—but doable—version of the experiment was identified. Feedback from the participant on difficulty/frustration level also was noted and considered during this process. Although both experimental parameters were adjusted, maximizing sentence length was prioritized over maximizing the number of response options. It should be noted that all target sentences throughout the study began with the name “Jane,” and therefore the first list of response columns always consisted only of the written word “Jane” (rather than a list of possible names to choose from).

For five PWA participants (PWA1, PWA2, PWA3, PWA4, and PWA5), this process resulted in modifications to both sentence length and number of response options presented (see Table IV for details), relative to the standard values. Two-word sentences contained a name and a verb; four-word sentences contained a name, verb, adjective, and object. When three-word sentences were attempted during determination of modifications, they contained a name, verb, and object. Each participant was required to complete ten practice trials in quiet correctly with their prescribed modifications before proceeding with the experimental task. Once the modifications for a given participant had been set, they were kept constant for that participant throughout the experiment.

**TABLE IV.** Experimental modifications for matched pairs. Non-modified sentences had a length of five words (of which four were key words) and provided eight response options per key word. Note that for *all* sentences, the first word (“Jane”) was given and therefore was not counted as a key word.

		Number of key words	Number of response options per key word
PWA1	C1	1	4
PWA2	C2	3	4
PWA3	C3	3	6
PWA4	C4	3	8
PWA5	C5	3	8

For one PWA participant (PWA6), a range of modifications was attempted; however, even when the sentence length was reduced to only two words (e.g., “Jane sold”) and the list of options was reduced to three, this participant was unable to consistently select the correct responses and was therefore deemed ineligible to continue the experiment. The precise reasons for the participant's difficulty with the task were not fully clear but likely involved notable impairments in reading and/or verbal working memory. Providing fewer than three options was not attempted.

### D. Application of frequency-specific gain

In addition to the task modifications described above, individualized frequency-specific gain was applied to all stimuli throughout the experiment, based on each individual participant's pure tone hearing thresholds, using the National Acoustic Laboratories (NAL-RP) gain procedure (Byrne *et al.*, 1991). Gain profiles were calculated and applied separately for the right and the left ear. The purpose of applying gain was to isolate the effects of aphasia on performance by controlling for hearing loss to the extent possible; it also effectively narrowed the differences in hearing profiles between the matched pairs of PWA and controls. Although the NAL-RP gain procedure does not aim to fully restore audibility, it is a widely used algorithm designed to balance amplification benefits with listener comfort.

### E. Procedures

During the experiment, participants were seated in front of a computer screen in a double-walled sound-treated IAC (Industrial Acoustic Corporation, North Aurora, IL) booth. The experimental task comprised two masker conditions, as well as two “microphone conditions.” The two masker conditions were speech maskers, designated “speech,” and speech-shaped, speech-envelope-modulated noise maskers, designated “noise.” The first microphone condition provided spatial cues through impulse responses measured from microphones mounted in the two ears of the Knowles Electronic Manikin for Acoustical Research, to approximate natural binaural cues, and was designated “KEMAR.” The second microphone condition achieved a spatially tuned response using impulse responses recorded from a 16-microphone array mounted across the top of the KEMAR manikin [e.g., review in Kidd (2017)] and was designated “BEAM.” The microphone array produces a sharply tuned attenuation curve at the higher frequencies and a progressively broader response at the lower frequencies [cf. Best *et al.* (2017), Fig. 1, p. EL370]. The algorithm used to implement beamforming has been described elsewhere (Desloge *et al.*, 1997; Greenberg *et al.*, 2003; Kidd *et al.*, 2013). All participants completed four adaptive tracks in each of the four possible microphone/masker combinations (KEMAR-speech, BEAM-speech, KEMAR-noise, and BEAM-noise). This allowed for direct calculation of the benefit provided by the BEAM microphone condition relative to the KEMAR microphone condition when the maskers consisted of speech, as well as when the maskers consisted of noise.

Across all conditions, target sentences began with the word “Jane” and were presented at a source position corresponding to 0° azimuth (i.e., directly in front of the listener). Maskers were always presented simultaneously with the target and with each other at source positions corresponding to ±60° azimuth. The onsets of each word were always aligned. Participants were instructed that they should always listen to the sentence starting with the word “Jane” and ignore the other talkers or noise. For each trial, three talkers from a pool of eight female talkers were chosen at random (without replacement for that trial): one for the target and one for each of the maskers. The next trial included an independent draw of talkers from the set of eight, again without replacement on that trial. Following the listening portion of each trial, participants used a mouse to select their answers on a GUI on the computer screen.

Masked SRTs in each condition were determined through the use of adaptive tracks, using a one-up, one-down procedure that estimates the 50% correct point^2^ on the psychometric function (Levitt, 1971). Within each adaptive track, the first trial was always presented at a TMR of 30 dB; TMRs for subsequent trials were based on participant performance. Each time the participant reported the entire sentence correctly, the TMR decreased by a designated step size, while each time the participant reported any part of the sentence incorrectly, the TMR increased by a designated step size. Each instance in which the TMR changed direction (from positive to negative, or vice versa) was coded as a reversal. The designated step size was 5 dB for the first three reversals, and 2 dB thereafter. The track was discontinued after nine reversals, and the TMRs of the last six reversals were averaged to determine a threshold estimate for that track. In order to avoid trials where maskers might be uncomfortably loud, the specific target and masker levels varied relative to each other from trial to trial as described above but were always presented at a fixed overall level of 75 dB (plus that participant's gain).

**FIG. 2.**
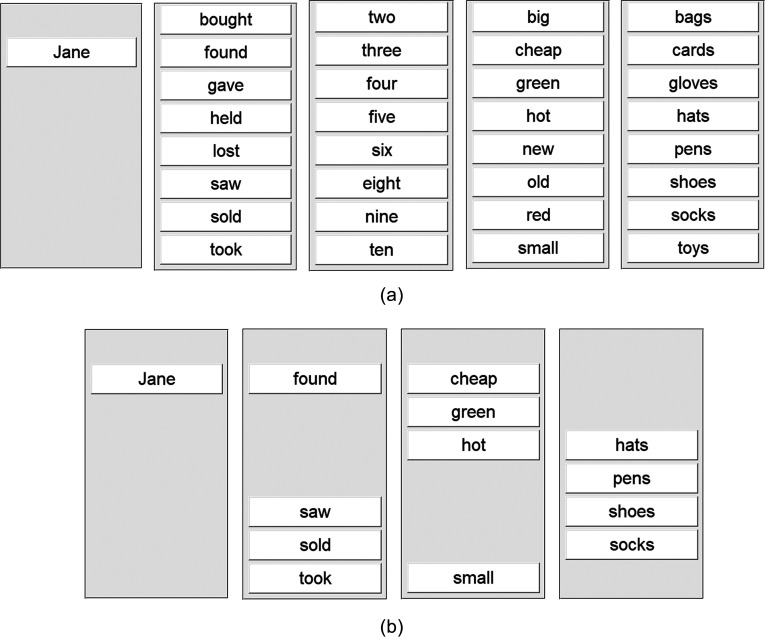
(a) Response GUIs presented (one by one) to controls in non-modified conditions. (b) Example modified response GUIs, presented one by one, in this case to PWA2 and C2.

Each PWA participant completed four adaptive tracks in each of the four combinations of microphone/masker conditions (for a total of 16 adaptive tracks). Each control participant was assigned^3^ to complete four adaptive tracks in each of the four combinations of microphone/masker conditions using *the same modified stimuli* as their PWA match (for a total of 16 adaptive tracks). Additionally, each control participant was also assigned to complete four adaptive tracks in each of the four combinations of microphone/masker conditions using *non-modified stimuli*^4^ (for a total of 16 *additional* adaptive tracks). Thus, control participants were assigned to complete the entire experimental task twice: once with their PWA match's modifications in place, and once with non-modified stimuli. The goal of this approach was to ascertain the effect of the modifications on controls' results. Please see Figs. 2(a) and 2(b) for examples of modified and non-modified response GUIs.

Throughout the experiment, the order of conditions was counterbalanced within and across participants. Additionally, for control participants, administration of modified and non-modified versions of conditions also was counterbalanced. Participants were encouraged to take breaks throughout the task as needed, and task administration was spread across multiple days for all participants in order to minimize fatigue.

## III. RESULTS

**FIG. 3.**
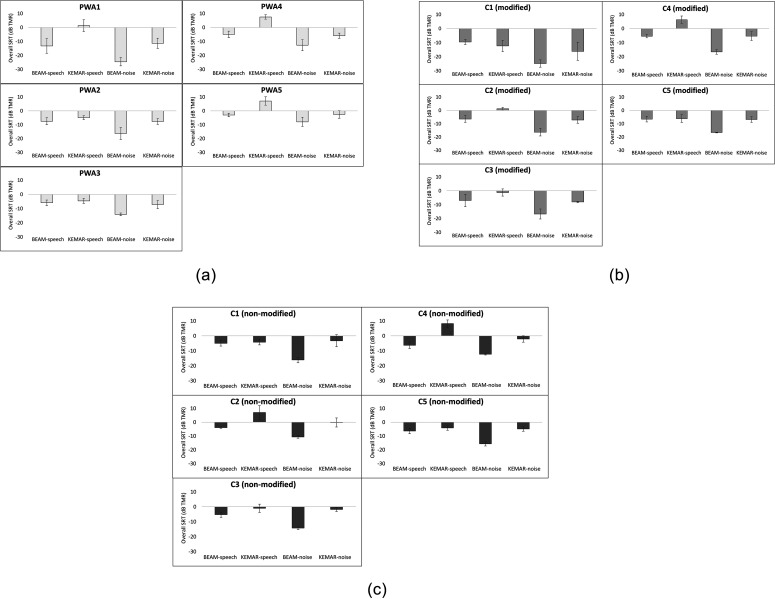
(a) Overall SRTs for each PWA participant in each microphone-masker condition. Error bars indicate standard deviations across estimates. (b) Overall SRTs for each control participant in each microphone-masker condition, in the modified version of the experiment. Error bars indicate standard deviations across estimates. (c) Overall SRTs for each control participant in each microphone-masker condition, in the non-modified version of the experiment. Error bars indicate standard deviations across estimates.

The four (or, in the aforementioned missing data cases, three) estimates for each participant in each microphone/masker condition were averaged to produce an overall SRT for that condition. The results from the individual participants are displayed in Figs. 3(a)–3(c). These figures permit comparison of BEAM vs KEMAR SRTs both within the speech masking condition and within the noise masking condition. Importantly, BEAM vs KEMAR SRTs for control participants may be compared within the set of modified conditions [Fig. 3(b)], as well as, separately, within the set of non-modified conditions [Fig. 3(c)].

Visual inspection of these results suggested that all participants exhibited substantially lower BEAM SRTs than KEMAR SRTs, indicating a benefit of the beamformer, in the noise masking condition. In the speech masking condition, however, only a subset of participants showed a similar benefit. Within the PWA group, three participants [PWA1, PWA4, and PWA5, see Fig. 3(a)] achieved substantially lower BEAM than KEMAR SRTs in the speech masking condition, indicating a benefit of the beamformer, while the remaining two PWA participants (PWA2 and PWA3) demonstrated similar BEAM and KEMAR SRTs in the speech masking condition, indicating no apparent benefit. Within the control group, three participants (C2, C3, and C4) also exhibited a benefit of the beamformer in the speech masking condition, whereas C1 and C5 did not. As will be discussed further below, each of these observations regarding the control participants held true for both the modified [Fig. 3(b)] and non-modified [Fig. 3(c)] sets of results.

It was also noted that all six participants who received a clear benefit from BEAM in the speech masking condition demonstrated positive (i.e., poorer) SRTs in the KEMAR-speech condition (or, in the case of C3, an SRT just under zero in this condition), while the four who did not benefit from BEAM in this condition demonstrated substantially negative (i.e., better) SRTs in the KEMAR condition.

**FIG. 4.**
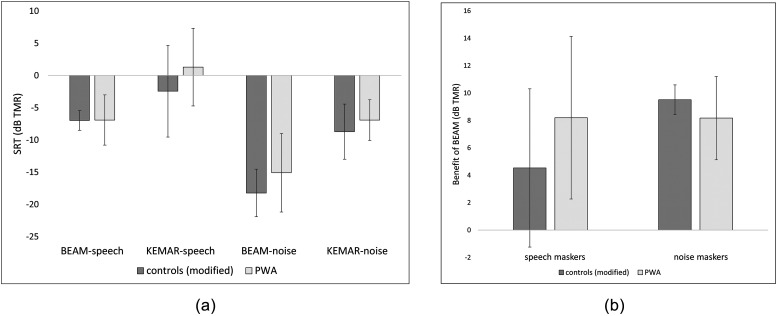
(a) SRTs in each microphone-masker condition, separated by group. SRTs from controls obtained using modified stimuli are shown. Error bars indicate standard deviations across participants. (b) Comparison of benefit of the beamformer for controls (modified stimuli) and PWA in each masking condition, calculated by subtracting BEAM from KEMAR SRTs. Error bars indicate standard deviations across participants.

Although the sample size of this study was small due to the complications of directly matching pairs of PWA and controls, the results were also examined at the group level. Figure 4(a) shows SRTs in each condition for PWA vs controls (modified). The group comparisons depicted in the Figure were evaluated using four independent-samples t-tests comparing PWA vs control SRTs in each microphone-masker combination (BEAM-speech, KEMAR-speech, BEAM-noise, and KEMAR-noise). All four yielded non-significant results (even without applying a correction of the significance level for multiple comparisons), indicating no meaningful group difference in SRTs. Figure 4(b) presents the same data as Fig. 4(a), but in a different configuration: the benefit of the beamformer (calculated by subtracting each participant's BEAM SRT from their corresponding KEMAR SRT) is shown for each group in both the speech masking and noise masking conditions.

**FIG. 5.**
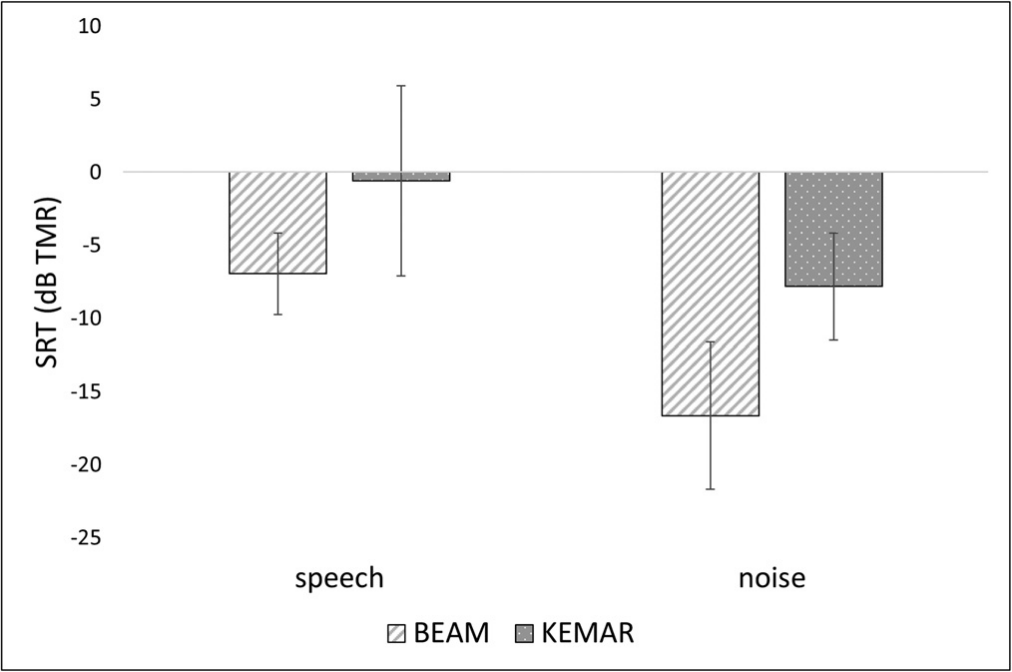
Comparison between BEAM and KEMAR SRTs in each masking condition for all participants as a single group. Error bars indicate standard deviations across participants.

The next analysis examined the effect of microphone condition and masking type on SRTs. Because the group comparisons reported above indicated there was no significant difference between SRTs for the PWA and control (modified) groups, the data from these two participant groups were combined for this analysis and treated as a single group of ten participants. A 2 × 2 repeated-measures analysis of variance was performed on these data to examine the effect of microphone condition and masking type on SRTs. Results indicated a significant main effect of microphone condition, F (1, 9) = 50.5, p < 0.001, as well as a significant main effect of type of masking, F (1, 9) = 118.6, p < 0.001, with no significant interaction effect (see Fig. 5 for group-level means).

**FIG. 6.**
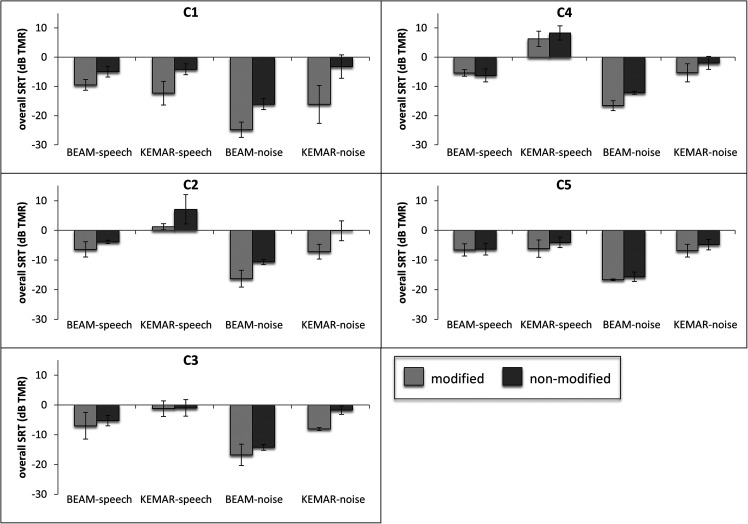
Comparison of SRTs on modified vs non-modified versions of the experiment for controls only. Error bars indicate standard deviations across estimates.

**FIG. 7.**
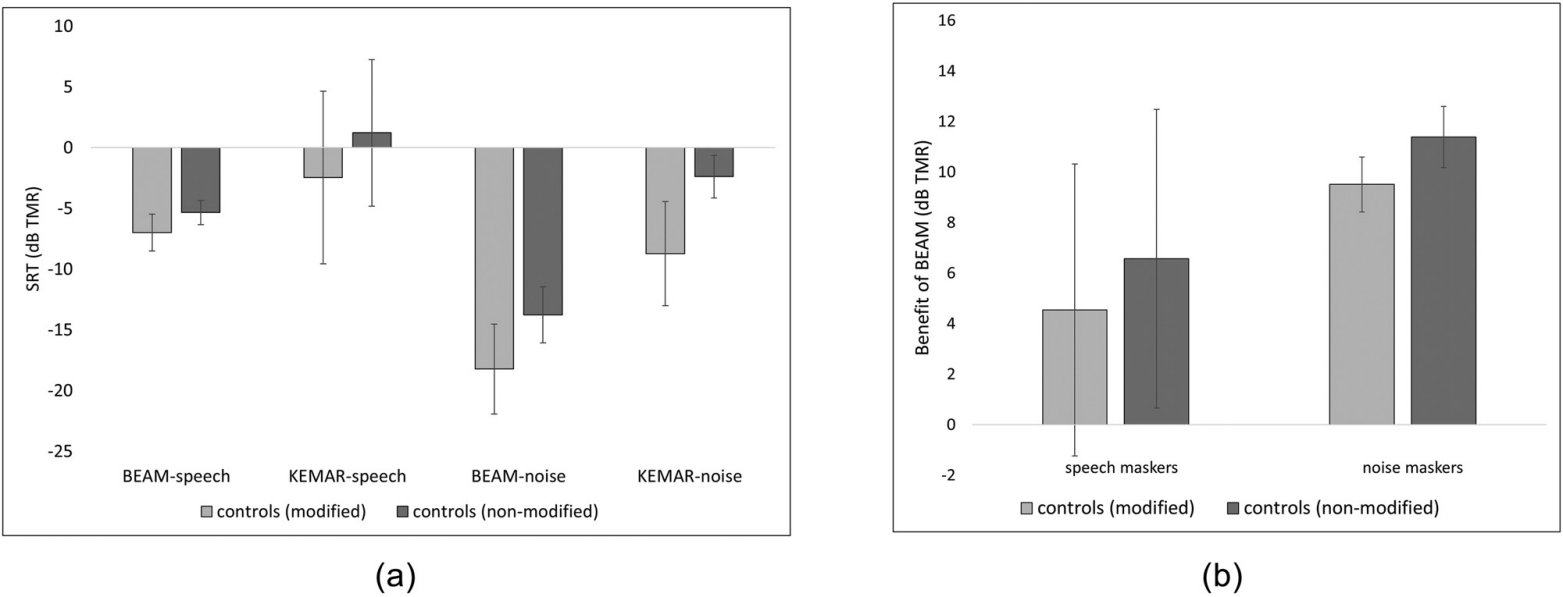
(a) Comparison of control SRTs obtained with modified vs non-modified stimuli, in each microphone-masker condition. Error bars indicate standard deviations across participants. (b) Comparison of benefit of the beamformer in each masking condition for controls with modified stimuli and controls with non-modified stimuli. Error bars indicate standard deviations across participants.

Because one of the aims of this study was to explore the feasibility of modifications to experimental methods to match the cognitive-linguistic abilities of PWA participants, understanding the effect of implementing these modifications was also of interest. Therefore, control results with modifications were compared to control results without modifications on the individual participant level (see Fig. 6). Visual inspection of these SRTs revealed that, while the precise thresholds differed between the modified and non-modified versions of the experiment for each of the control participants, the overall patterns remained the same. For instance, participants who received a benefit in the speech masking condition (C2, C3, and C4) received this benefit in both the modified and non-modified versions of the experiment; likewise, participants who obtained no benefit from the beamformer in the speech masking condition obtained no benefit in both the modified and non-modified versions. Additionally, all participants obtained a benefit of the beamformer in the noise masking condition, both in the modified and non-modified versions. These comparisons were examined at the group level as well, where similar patterns were noted [see Figs. 7(a) and 7(b)].

**FIG. 8.**
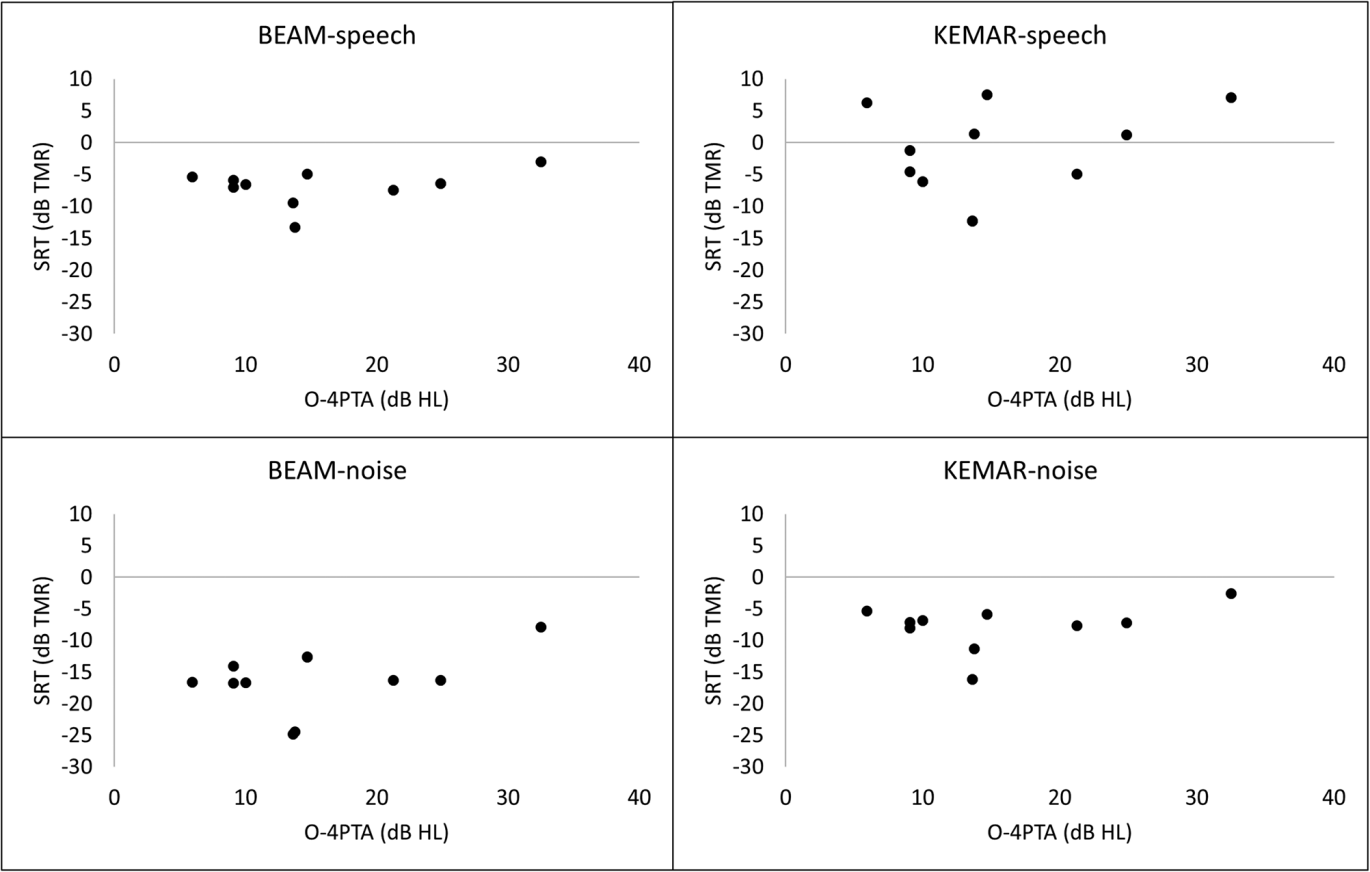
Relationships between pure tone averages and SRTs in each microphone-masker condition (all participants considered as a single group; control data are from the modified versions of the experiment).

Finally, the relationship between participants' hearing profiles and their performance on the experimental task was examined in order to determine whether degree of hearing loss may have influenced results. Because frequency-specific gain was added to all stimuli based on participants' hearing profiles in order to help compensate for hearing loss and ensure that the stimuli were audible, no relationship between the audiometric profile and the experimental results was expected. For this analysis, data from all ten participants who completed the experimental task were examined as a single group. An overall 4-frequency pure-tone average (O-4PTA) was calculated for each participant by first finding the mean of their pure-tone thresholds (in dB hearing level) at 500 Hz, 1 kHz, 2 kHz, and 4 kHz both for the right ear and for the left ear, and then finding the mean of those two numbers. Participants' O-4PTAs were then plotted against their SRTs in each of the four conditions (see Fig. 8). No relationships were observed, and each of four Pearson correlation analyses examining these relationships produced a non-significant result.

## IV. DISCUSSION

This study investigated the benefit of acoustic beamforming for improving speech recognition in persons with aphasia and age- and hearing-matched controls, under both high-informational masking and high-energetic masking conditions. The study also examined whether individually tailored modifications to experimental procedures could make typical psychoacoustic/speech identification methods appropriate for use with listeners with aphasia. The results provided evidence that acoustic beamforming may indeed be useful for facilitating speech recognition in complex, multiple-source listening situations both for listeners with aphasia and for age- and hearing-matched controls, under high-EM conditions where maskers consist of speech-shaped, speech envelope-modulated noise that is spatially separated from the target. There was also evidence that beamforming may be useful for improving speech recognition under spatially separated high-IM conditions for some listeners in each group; however, the extent of this benefit may depend on how well a listener is able to perform under such conditions without the beamformer. This finding is qualitatively similar to past work comparing the benefits of acoustic beamforming for listeners with normal hearing and with sensorineural hearing loss [e.g., Kidd *et al.* (2015)]. Additionally, the results indicated that the types of adaptations of psychoacoustic methods that were implemented in this study were feasible and did not appear to affect the overall patterns of performance, suggesting that they may be useful for obtaining measurements of masked speech recognition thresholds in PWA, as well as in other populations of listeners with cognitive-linguistic impairments.

Due to the lack of a visible (or statistically supported) difference between PWA and control SRTs with modified stimuli, our group-level analysis investigating the effect of the beamformer treated all participants as members of a single group drawn from a population that might best be characterized as “older listeners with mild hearing loss, with or without aphasia.” This group-level analysis revealed that the beamformer resulted in significantly lower SRTs than did natural spatial cues. This result indicates that, overall, participants in the current study did benefit from implementation of the beamformer. We consider this finding to provide support for the idea that beamforming could be a useful approach in improving masked speech recognition across a variety of populations for whom a hearing aid fitting normally might not be regarded as an option. The extent to which beamforming may be useful, however, may depend on masker type as well as on individual listener characteristics and abilities, as discussed in the following paragraphs.

Visual inspection of individual-level results provided additional information about the utility of the beamformer under each of the two types of masking conditions examined in this study. Specifically, it was observed that the beamformer facilitated a decrease in SRT (relative to the “KEMAR” listening condition which provided binaural spatial cues) for all PWA and control listeners in the high-EM, low-IM condition where maskers consisted of speech-shaped, speech envelope-modulated noise, indicating that they received a benefit of the beamformer in this condition. This result is not surprising, as the main consequence of using beamforming in a high-EM condition is an improved TMR, which typically results in lower (better) SRTs relative to natural listening situations for most listeners, whether normal hearing or hearing impaired (Kidd, 2017). Because the beamformer presents sounds to the listener through a single channel, the binaural spatial cues that would be available in naturalistic listening situations are not present. However, any negative effect of the absence of spatial cues in the beamforming condition was outweighed by the benefits of the improved TMR for the tasks used here. This finding likely occurred because spatial cues—especially when head shadow effects are minimized by symmetric placement of maskers around the target—are generally less effective for separating speech from noise (i.e., in reducing EM) than they are for perceptually segregating target speech from competing speech (i.e., in reducing IM).

Additionally, visual inspection of the individual results suggested that, for six of the ten listeners, the beamformer also provided a benefit in the high-IM, low-EM condition, where maskers consisted of intelligible speech similar to the target speech, whereas for the remaining four participants, this benefit was not seen. As discussed earlier, prior work from our group has indicated that while beamforming generally provides a benefit in high-IM conditions for listeners with SNHL when there is substantial spatial separation between target and masker, it can actually result in higher (poorer) SRTs for some normal-hearing listeners under the same conditions (Kidd *et al.*, 2015). Therefore, the effect of the beamformer for six of the listeners in the current study was similar to the effect previously observed for listeners with SNHL. For the remaining four listeners in the current study, beamforming did not seem to have either a positive or negative impact on performance. Like the current study, Kidd *et al.* (2015) applied frequency-specific gain for listeners in the SNHL group. However, there were also some key differences between the listeners in the current study and the listeners in the 2015 study. The listeners in the current study had less hearing loss overall than the SNHL listeners in the earlier study, which could have affected not only their listening abilities and strategies but also how much benefit the gain provided. Additionally, the listeners in the current study were substantially older than the SNHL listeners in the earlier study, and masked speech reception thresholds are known to increase with age (Gallun *et al.*, 2013; Dubno, 2015). These results underscore previous findings that while the beamformer can provide a benefit in high-IM spatially separated listening conditions, this may not be true for all listeners.

Further examination of individual results provided valuable clues about who may benefit most from the beamformer and why. The factor that seems to best predict whether an individual participant will benefit from the beamformer in the speech masking condition is how well that participant performed in the speech masking condition *without* the beamformer, i.e., in the KEMAR condition that provided binaural spatial cues. All six of the listeners in the current study who benefited from the beamformer had positive SRTs in the KEMAR-speech condition (or, in the case of C3, only a slightly negative SRT in the KEMAR-speech condition), indicating a relatively poor ability to use binaural spatial cues to segregate target speech from masker speech. It is even possible that some of these listeners may have relied on a level cue to identify and attend to the target talker—i.e., the simple fact that the target talker was louder than the masker talkers may have enabled these listeners to perceptually segregate the target [see discussion of this effect in Brungart *et al.* (2001) and Kidd *et al.* (2015)]. Interestingly, there is also evidence that in high-IM listening conditions, use of a level cue to segregate target from colocated maskers may require lower perceived effort than exploiting binaural cues in spatially separated conditions (Rennies *et al.*, 2019).

In contrast, the four listeners who did not appear to receive a benefit of the beamformer all achieved clearly negative SRTs in the KEMAR-speech condition, indicating that they possessed some ability to take advantage of binaural spatial cues to isolate the target. There was no evidence that any other factor (such as pure tone detection thresholds, or, for PWA participants, aphasia type or severity) was related to performance with respect to susceptibility to IM. We therefore tentatively conclude that older listeners with mild hearing loss (again, with or without aphasia) who are poor at using spatial cues to separate target speech from masker speech in a naturalistic listening condition providing binaural spatial cues may benefit from acoustic beamforming, likely because it provides a boost in TMR which enables them to segregate the talkers based on a level cue [in addition to using cues available from identification of the target talker's voice; e.g., Best *et al.* (2018)], but that beamforming does not provide an additional benefit for listeners who are able to use spatial cues effectively.

**FIG. 9.**
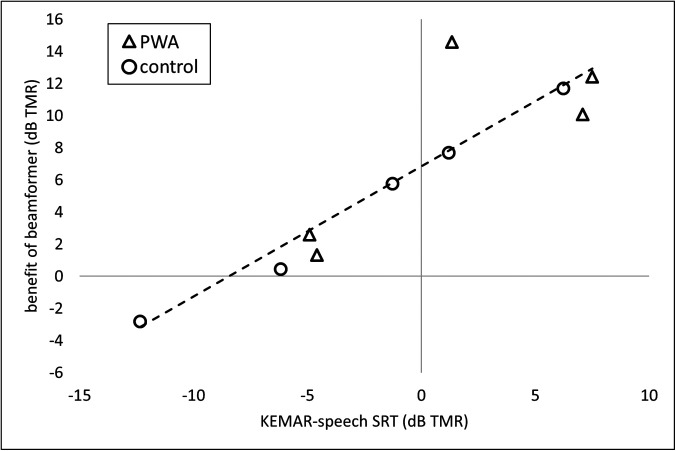
Benefit of the beamformer in the speech masking condition (calculated by subtracting each participant's BEAM-speech SRT from their KEMAR-speech SRT), plotted as a function of KEMAR-speech SRTs.

Evidence of a similar pattern has been observed in earlier work as well: Kidd *et al.* (2015) found that within a group of NH listeners (N = 8), the seven listeners who were able to successfully take advantage of binaural spatial cues to achieve low SRTs in a KEMAR speech-on-speech masking condition demonstrated *poorer* performance when the beamformer was provided, whereas the sole NH listener whose SRT in the KEMAR condition was poorer (around zero) improved when the beamformer was provided. A similar trend was also observed within the SNHL listener group in that study. This pattern was conceptualized as a possible association whereby the worse a listener performed in a KEMAR speech-masking condition, the more benefit they received from the beamformer in this masking condition [Kidd *et al.* (2015), Fig. 5, p. 8]. Visual examination of data from the current study suggests a similar associative relationship, where a higher (poorer) KEMAR-speech SRT is associated with a greater benefit of the beamformer (see Fig. 9; note, however, that no statistical correlation analysis was performed due to the lack of independence between the two variables).

Our decision to examine PWA and controls as a single group within the context of the current study should not be interpreted as an argument that these two groups are equivalent or that aphasia has no impact on speech recognition abilities. On the contrary, our earlier (non-beamforming) study examining SRTs in two similar groups of participants found that that PWA, as a group, achieved poorer SRTs than controls under high-IM masking conditions where target and maskers were spatially separated (Villard and Kidd, 2019). While the KEMAR-speech SRTs obtained in the current study were broadly consistent with this previous finding (i.e., the group mean KEMAR thresholds were higher for PWA than controls and the group mean benefit was larger), the group differences here were not statistically significant. Both studies observed substantial variability in SRTs in both groups under high-IM listening conditions. In fact, in the previous study, the significant group difference was only observable with a larger sample size of 12 participants in each group. The lack of a clear group difference in the current study, therefore, could be due to the smaller sample size. It should also be noted that the existence of person-to-person variability in performance on speech-on-speech listening tasks was not unexpected and has been documented under similar high-IM listening conditions in a number of previous studies, e.g., Clayton *et al.* (2016), Swaminathan *et al.* (2015), and Oberfeld and Kloeckner-Nowotny (2016).

In comparing the current study to our 2019 study (Villard and Kidd, 2019), it is also interesting to note that there are clear quantitative differences between their mean SRTs, both for PWA and for controls. The current study's speech-on-speech masking condition involving binaural spatial cues (KEMAR-speech) elicited a mean SRT of 1.3 dB TMR for PWA and −2.5 dB TMR for controls (modified). In the 2019 study, a similar condition elicited lower mean SRTs: −2.4 dB TMR for PWA and −14.0 dB TMR for controls. The same was true for a condition where binaural spatial cues were provided but speech was masked by noise: in the current study, the mean SRT in this condition (KEMAR-noise) was −6.9 dB TMR for PWA and −8.7 dB TMR for controls (modified), whereas in the 2019 study the corresponding mean SRTs were −15.0 dB TMR and −17.0 dB TMR, respectively. Additional insights about these differences were also available through direct comparisons of SRTs on the individual level, as eight of the ten participants in the current study (all five PWA, as well as three controls) had also participated in the 2019 study. These individuals' SRTs from each of the two studies are presented side by side in Table V. In general, these differences were consistent with the differences between the group means, in that the SRTs in the current study were higher (poorer) than those in the earlier study.

**TABLE V.** Comparisons between SRTs for individual listeners in the current study vs in our earlier study. Control data from the current study are from the modified versions of the experiment.

	Speech masking SRTs (dB TMR)			Noise masking SRTs (dB TMR)		
	Current study	Villard and Kidd (2019)	Difference	Current study	Villard and Kidd (2019)	Difference
PWA1	1.3	4.8	3.5	−11.3	−19.2	−7.9
PWA2	−4.9	−17.2	−12.3	−7.7	−14.9	−7.2
PWA3	−4.6	−14.0	−9.4	−7.2	−13.9	−6.7
PWA4	7.5	−10.9	−18.4	−5.9	−15.7	−9.8
PWA5	7.1	0.4	−6.7	−2.6	−13.5	−10.9
C1	−12.3	*n/a*	*n/a*	−16.2	*n/a*	*n/a*
C2	1.2	−14.0	−15.2	−7.2	−17.2	−10.0
C3	−1.3	−14.3	−13.1	−8.1	−16.1	−8.0
C4	6.3	*n/a*	*n/a*	−5.3	*n/a*	*n/a*
C5	−6.2	−19.1	−12.9	−6.8	−17.4	−10.6

While it may not be possible to definitively identify the reasons why the current study elicited poorer SRTs than the 2019 study in both these conditions, we suggest that these differences were likely driven by differences in the experimental task and stimuli. Stimuli in the earlier study were confined to a closed set of only four nouns (only one of which was presented on each trial), and response options always consisted of four pictures of these same nouns, resulting in a relatively high amount of predictability from trial to trial, as well as a relatively low demand on working memory. In contrast, the current study asked participants (with the exception of PWA1 and C1 in their modified version of the experiment) to recognize, recall, and respond to multiple words—including verbs and adjectives—on each trial. Furthermore, even when response options were reduced to fewer than eight per trial, the words presented on each trial were still drawn from the full set of eight, increasing the listener's uncertainty about what might be presented next. Increasing the number of words to be attended to and remembered on a given trial, as well as increasing the size of the closed set from which they were drawn, would be expected to increase overall task demands, possibly resulting in higher (poorer) SRTs—and indeed, this is what was observed. We hypothesize that if task demands had been more similar between the two studies, SRTs for similar conditions would have been more quantitatively similar as well. Finally, response selection in the current study involved words rather than pictures, which could have impacted the response process in unknown ways.

While the available literature on masked SRTs in PWA is limited to our own previous study, it may be useful to compare the SRTs obtained for controls in the current study to SRTs obtained in previous work on speech-on-speech masking conditions in older listeners with normal hearing or mild hearing loss. One study found that listeners whose ages were comparable to those in the current study achieved 50% correct SRTs between approximately −8 and −3 dB TMR (Gallun *et al.*, 2013), another study found them to be between approximately −5 and −3 dB TMR (Marrone *et al.*, 2008), and a third study measured them between approximately −6 and −4 dB TMR (Helfer and Freyman, 2008). All three of these earlier studies, therefore, found SRTs that were somewhat better than the SRTs obtained in the KEMAR-speech condition in the current study (−2.5 dB TMR with modified stimuli and 1.2 dB TMR with non-modified stimuli). It is not possible to conclude from our data why the control SRTs in the current study are poorer than would have been predicted based on these previous findings. However, as noted above, it is typical to see substantial person-to-person variability in performance on speech-on-speech listening conditions; therefore, the unexpected SRTs in the current study could be attributable in part to its relatively small sample size.

Results from the current study suggest that the individualized modifications to experimental stimuli made the experiment accessible to five of the six PWA initially enrolled, allowing for the measurement of SRTs in different conditions without noticeable confounds related to impaired comprehension, working memory, reading, scanning, or strategy use in these participants. Observations during the determination of participants' modifications suggest that these confounds would likely have substantially impacted performance if the experiment had been conducted with the five-word sentences and lists of eight response options typically employed in this task. With the modifications in place, performance during the initial trials of the adaptive tracks declined as expected, suggesting that PWA were able to successfully complete early trials and only encountered difficulty as TMRs began to decrease.

Because each control participant completed both a modified and non-modified version of the experiment, we were able to directly examine the effect of each set of modifications on SRT. An immediate effect of reducing sentence length and reducing number of options presented is that the chance of guessing an entire sentence correctly increases. With non-modified stimuli, for example, a participant would have only a 1-in-8 chance of guessing correctly on a given word, and a 1-in-4096 chance of guessing correctly on a given trial (i.e., guessing an entire sentence correctly). In contrast, PWA1—as well as C1 in the modified version of the experiment—were presented with two-word sentences (only one of which was scored, as the first word, “Jane,” was always given) and four response options and therefore had a 1-in-4 chance of producing a correct sentence with a random guess. This increased chance of guessing correctly would be expected to result in a higher proportion of correct responses at lower TMRs, which in turn would result in a decrease in 50% correct SRT. The other sets of modifications provided participants with intermediary probabilities of guessing a single trial correctly: PWA2 and C2 had a 1-in-64 chance of guessing correctly with modifications in place, PWA3 and C3 had a 1-in-216 chance, and the remaining participants had a 1-in-512 chance. It may be relevant to note that, in comparing the SRTs obtained during the current study with those obtained for the same individuals who also participated in our earlier study (Villard and Kidd, 2019), the participant whose SRTs were most similar between the two studies was PWA1. This was the PWA participant who required the greatest degree of modification in the current study and, consequently, had a 1-in-4 chance of guessing correctly on a given trial. This, incidentally, was the same chance that all participants had of guessing correctly in the 2019 study, where each trial involved selection of a single response from a field of four presented pictures. This could indicate that the chance of guessing correctly played a role in determining participants' SRTs in the current study; however, PWA1 is the only participant for whom this particular comparison is possible, as his matched control (C1) did not participate in the earlier study.

While an increased probability of lucky guesses may help explain why participants' SRTs were lower when the modifications were in place, there are several reasons why this should not be accepted as the sole explanation. To begin with, the theory that participants take random guesses on trials where the correct answer is not known to them may not adequately take into account features of the experiment that are endemic to the study of informational masking, such as masker-based errors [e.g., Brungart *et al.* (2001)]; the likelihood of such errors may vary depending on TMR. Additionally, as discussed earlier, implementation of the modifications may simply have rendered the modified trials less taxing overall, resulting in lower SRTs. Even though the control participants—unlike the PWA participants—were able to complete the non-modified version of the experiment successfully, the task of listening to five-word sentences and choosing from lists of eight response options may have placed a substantial burden on their cognitive resources (particularly working memory), resulting, perhaps, in poorer overall performance and increased TMRs. Therefore, while our data do not allow us to pinpoint exactly why SRTs decreased across the board when modifications were implemented, it seems reasonable to conclude that these decreases were due to some combination of factors relating to experimental stimuli and/or parameters.

That being said, perhaps the most notable finding from the current study's comparison of modified and non-modified versions of the experiment in control listeners is that while implementation of the modifications did generally lower TMRs, the overall patterns of performance remained the same for each control participant, regardless of whether or not the modifications were in place. The participants whose TMRs were poor in the KEMAR-speech condition but improved in the BEAM-speech condition (C2, C3, and C4) showed this result in both versions of the experiment. Similarly, the participants whose TMRs were already good in the KEMAR-speech condition and did not show a further improvement in the BEAM-speech condition (C1 and C5) also showed this same pattern in both versions. And all five control participants demonstrated a clear benefit of the beamformer in the noise masking condition, in both the modified and non-modified versions. The conclusion we draw from these results, therefore, is that these or similar modifications may be a promising strategy for removing experimental confounds in psychoacoustics research with special populations. On a related note, other work examining the role of set size (i.e., number of response options) in psychoacoustic experiments suggests that adjusting set size for different listeners based on listening ability may be able to remove other types of confounds such as the effects of dips in a fluctuating masker at various TMRs (Bernstein *et al.*, 2012).

Notably, the specific modifications used in the current study—reducing sentence length and reducing number of written word response options—may not be sufficient to render psychoacoustic experiments accessible to all PWA. One participant enrolled in the current study (PWA6) was unable to complete the experiment due to his difficulty reporting target sentences in quiet, even with the maximum number of modifications in place. Therefore, while these modifications may enable a certain subset of PWA to undergo psychoacoustic testing, others with more severe impairments may require a different experimental structure in order for SRTs to be measured. As discussed above, for example, our earlier study (Villard and Kidd, 2019) used pictured response options and only asked participants to recall the last word of a sentence. While that study's approach may introduce some complications, it does appear to have the advantage of enabling measurements of SRTs for individuals whose speech reception abilities cannot be reliably tested by traditional means.

One final observation concerns the limitations of the benefits of a single channel beamformer that eliminates spatial perception of sound sources and, consequently, reliable sound source localization. Although it was outside of the scope of the current study, there are a variety of approaches in the literature that attempt to preserve spatial hearing while affording the improvement in TMR from beamforming. These efforts include a hybrid natural-beamformer solution termed “BEAMAR” (Kidd *et al.*, 2015; Best *et al.*, 2017), an algorithm that imposes beamforming on glimpsed stimuli processed according to criterion interaural differences (Wang *et al.*, 2020) and a “triple beam” algorithm [Jennings and Kidd (2018); see also Yun *et al.* (2019)] that adds left-ear- and right-ear-only beams focused to the sides of the primary target-focused beam. All of these approaches preserve some degree of sound source localization and have strengths and weaknesses that may carry different weights for deciding on the best algorithm for persons with specific underlying complaints (e.g., SNHL vs deficits in selective attention or cognitive-linguistic functional abilities). Further study of these algorithms under a range of conditions appears to be warranted.

Results from this study suggest that a beamforming hearing aid can be beneficial in cocktail party listening situations for a wide range of listeners, including older persons with different variations of mild hearing loss, either with or without aphasia. More specifically, acoustic beamforming may be beneficial for improving speech reception thresholds in PWA and controls under high-EM masking conditions, as well as under high-IM masking conditions for some listeners, particularly those who are poor at using spatial cues to separate target and masker talkers. Notably, our findings suggest that a spatially tuned assistive listening device or hearing aid may be beneficial when the primary complaint is *not* hearing loss but rather selective attention or cognitive-linguistic deficits. Additionally, results suggest that experimental modifications may help make psychoacoustic experiments appropriate for use with PWA and that while the modifications used in this study did have an effect on specific TMRs, they did not appear to have a meaningful effect on overall patterns of performance or comparisons between TMRs in different experimental conditions.

## V. CONCLUSION

Persons with aphasia have been shown to exhibit difficulty understanding speech in complex acoustic environments, necessitating the development of possible compensatory and/or rehabilitative strategies to ameliorate this issue. The current study investigated the effectiveness of acoustic beamforming in listeners with aphasia and found that beamforming may be an effective tool for these listeners under certain conditions, as well as for listeners of similar age and hearing status who do not have aphasia; however, it also indicated that the benefit provided by the beamformer may depend on the type of masker present as well as on individual listener characteristics. This study also tested several possible experimental modifications that may render psychoacoustic paradigms more accessible to individuals with aphasia; results suggest that certain modifications of stimuli may allow for accurate measurement of listening abilities in this population. Future work should examine the utility of modifications in a larger group of participants and should more closely examine the impact of these modifications on performance.

## Footnotes

^1^ “Clinical judgment” in this case refers to the observations regarding the functional ability of PWA to successfully complete the task and the matching of task complexity to those abilities. These decisions are referred to as “clinical” because they are similar to the judgments clinicians routinely make in matching task demands—either diagnostic or therapeutic—to clients' capabilities. It is acknowledged that, for this research application, other clinicians might have chosen to make different modifications for these participants.

^2^ The choice of tracking the 50% correct point on the psychometric function was based on the advantages of simplicity and consistency across participants. In making this decision, we recognized that the point designated as “threshold” would fall at different places on the psychometric functions depending on the chance point associated with the modifications made to accommodate the PWA participants and correspondingly used with the control participants.

^3^ Due to unforeseen and unavoidable circumstances near the end of the data collection period, three control participants were not able to complete 100% of their assigned adaptive tracks, resulting in a small amount of missing data. Specific instances of missing data for controls are as follows: C1 completed only three adaptive tracks in the KEMAR-speech condition with modified stimuli and only three adaptive tracks in the KEMAR-noise condition with non-modified stimuli. C2 completed only three adaptive tracks across conditions with modified stimuli. C4 completed only three adaptive tracks in the BEAM-noise condition with non-modified stimuli. All other participants completed all tracks as planned.

^4^ “Non-modified stimuli” means that the full experimental matrix (see Table III) was used: participants were presented with five-word sentences and were give the full set of eight possible response options for each word.
